# Biomimetic nanobubbles for triple-negative breast cancer targeted ultrasound molecular imaging

**DOI:** 10.1186/s12951-022-01484-9

**Published:** 2022-06-10

**Authors:** Natacha Jugniot, Tarik F. Massoud, Jeremy J. Dahl, Ramasamy Paulmurugan

**Affiliations:** 1 Molecular Imaging Program at Stanford (MIPS), and Bio-X Program, Department of Radiology, School of Medicine, Stanford University, Stanford, CA 94305-5427 USA; 2 Canary Center at Stanford for Cancer Early Detection, Department of Radiology, School of Medicine, Stanford University, Stanford, CA 94305-5427 USA; 3 Molecular Imaging Program at Stanford (MIPS), Canary Center for Cancer Early Detection at Stanford, Stanford University School of Medicine, 3155 Porter Drive, Palo Alto, CA 94304 USA

**Keywords:** Triple negative breast cancer, Nanobubble, Molecular imaging, Cancer early detection, Cancer cell membrane, Ultrasound (US), Homotypic targeting

## Abstract

Triple-negative breast cancer (TNBC) is a highly heterogeneous breast cancer subtype with poor prognosis. Although anatomical imaging figures prominently for breast lesion screening, TNBC is often misdiagnosed, thus hindering early medical care. Ultrasound (US) molecular imaging using nanobubbles (NBs) capable of targeting tumor cells holds great promise for improved diagnosis and therapy. However, the lack of conventional biomarkers in TNBC impairs the development of current targeted agents. Here, we exploited the homotypic recognition of cancer cells to synthesize the first NBs based on TNBC cancer cell membrane (i.e., NB_CCM_) as a targeted diagnostic agent. We developed a microfluidic technology to synthesize NB_CCM_ based on the self-assembly property of cell membranes in aqueous solutions. In vitro, optimal NB_CCM_ had a hydrodynamic diameter of 683 ± 162 nm, showed long-lasting US contrast enhancements and homotypic affinity. In vivo*,* we demonstrated that NB_CCM_ showed increased extravasation and retention in a TNBC mouse model compared to non-targeted NBs by US molecular imaging. Peak intensities and areas under the curves from time-intensity plots showed a significantly enhanced signal from NB_CCM_ compared to non-targeted NBs (2.1-fold, *P* = 0.004, and, 3.6-fold, *P* = 0.0009, respectively). Immunofluorescence analysis further validated the presence of NB_CCM_ in the tumor microenvironment. Circumventing the challenge for universal cancer biomarker identification, our approach could enable TNBC targeting regardless of tumor tissue heterogeneity, thus improving diagnosis and potentially gene/drug targeted delivery. Ultimately, our approach could be used to image many cancer types using biomimetic NBs prepared from their respective cancer cell membranes.

**Supplementary Information:**

The online version contains supplementary material available at 10.1186/s12951-022-01484-9.

## Introduction

Accounting for 15–20% of all breast carcinomas, triple-negative breast cancer (TNBC) is defined by the absence of estrogen receptor (ER), progesterone receptor (PR), and human epidermal growth factor 2-receptor (HER2) [1]. Compared to hormone receptor-positive and HER2-positive breast malignancies, TNBC has a more aggressive clinical course with faster growth rate, higher risk of metastasis and recurrence, yet without targeted therapies. Moreover, the molecular profiling of TNBC has revealed a high level of histopathological, transcriptomic, and genomic heterogeneity, making diagnosis challenging [2].

Currently, mammography is the first-line method for breast cancer (BC) diagnosis. However, malignant lesions can be rendered radiographically hidden by dense or heterogenous breast tissues or they can appear as benign fibroadenomas [3]. Consequently, supplemental imaging modalities, such as magnetic resonance imaging (MRI) and ultrasound (US), are frequently employed to further diagnose suspicious breast lesions. Although MRI is useful in screening high-risk patients, this modality is not widely available, typically requires long scanning times, and it has some contraindications. Abbreviated MRI and ultrafast sequences have emerged to make MRI cheaper and faster, but both sensitivity and specificity were diminished in multiple studies [4–6].

With greater accessibility and lower cost, US is a more compatible imaging modality for widespread BC screening. Contrast-enhanced US (CEUS) has been appealing owing to its potential for cancer molecular imaging. CEUS takes advantage of highly echogenic ultrasound contrast agents (UCAs) producing unique non-linear signals [7]. Currently, gas-filled micron-sized bubbles (MBs) are strict vascular UCAs that permit assessment of blood flow and vascular density. The introduction of molecularly targeted MBs that bind to vascular markers expressed in diseased tissues constitutes a major advancement in cancer early detection, characterization, and therapy [8]. Recently, the first targeted MB (BR55) has received Investigational New Drug approval from the US Food and Drug Administration to allow further clinical testing (NCT02142608) for breast lesions [9]. Interestingly, BR55 showed a signal enhancement in 93% of malignant breast lesions compared to 33% in benign breast lesions. To more effectively differentiate between malignant and benign lesions, nanobubbles (NBs) have been introduced for their capacity to target extravascular markers [10]. Smaller in size, NBs can accumulate in the tumor microenvironment through passive targeting based on the enhanced permeability and retention effect (EPR) [11]. Furthermore, when conjugated to a specific ligand, NBs can also actively target oncogenic markers, further enhancing the US signal. Currently, NBs targeting HER2 [12, 13], prostate specific membrane antigen (PSMA) [14, 15] and cancer antigen 125 (CA-125) [16] have been investigated in vivo for breast, prostate, and ovarian cancer diagnosis, respectively. However, as TNBC lacks unique surface markers, conventional targeted diagnosis strategies have been unsuccessful [12]. In this regard, novel approaches in the design and formulation of targeted UCAs are essential.

**Scheme 1.**
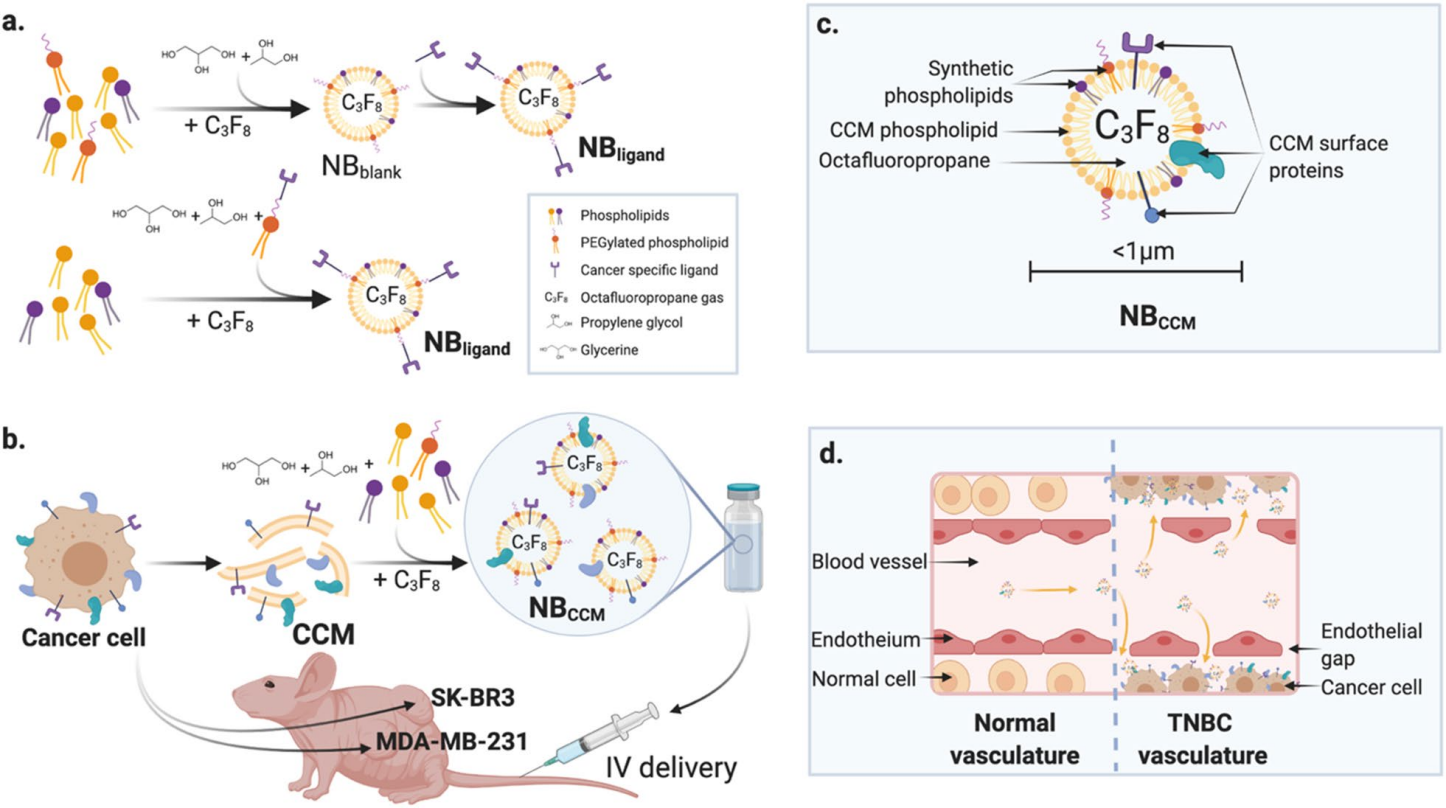
Tumor-targeted NBs. **a** Conventional targeted NBs produced by lipid film hydration functionalized with a cancer specific ligand (NB_ligand_). The ligand can be conjugated on a pre-formed bubble (top), or, to one element constituting the shell before bubble formation (bottom); **b** CCM-based NB for US molecular imaging of TNBC developed in this study, with **c** its representative structure, and **d** its mechanism of tumor targeting across endothelial junctions

A prominent strategy for TNBC targeting exploits the unique feature of tumor cells referred to as homotypic recognition (i.e., recognition and binding between cancer cells) [17, 18]. Although the mechanism remains unclear, cancer cell membrane (CCM) surfaceome is equipped with cell–cell and cell-extracellular matrix adhesion molecules that could participate in homotypic targeting [19, 20]. Interestingly, the downregulation of these cell adhesion molecules (CAMs) could participate in tumor cell translocation and metastasis [21, 22]. Hence, the inherent property of CCMs for strong homotypic adhesion has been investigated for tumor targeting, and many studies have focused on CCM-coated nanoparticles for targeted delivery of therapeutics [23–27]. Nonetheless, to our knowledge no CCM-based UCAs have been examined to date. In this study, we introduce a new archetype of NBs constructed from CCMs for TNBC diagnosis. We used a microfluidic device comprising a T-junction architecture to produce uniform NBs by a pressure-based disruption and reconstitution process. We hypothesized that NB_CCM_ would provide a complete replication of membrane-associated proteins from the source cells onto the engineered NBs. A summary of the study design is presented in Scheme 1. This approach could circumvent the challenge of synthetically replicating natural cell surfaces while maximizing tumor targeting regardless of tissue heterogeneity. Overall, we demonstrated a successful active targeting of TNBC using NB_CCM_ and established its superiority compared to non-targeted NBs both in vitro and in a TNBC mouse model. Our results highlight the diagnostic ability of CCM-based NBs. This personalized medicine approach could allow a minimally invasive and highly accurate TNBC diagnosis and would be especially useful in high-risk patients who cannot undergo MRI exams.

## Results

### Preparation and characterization of CCM-based NBs

**Fig. 1:**
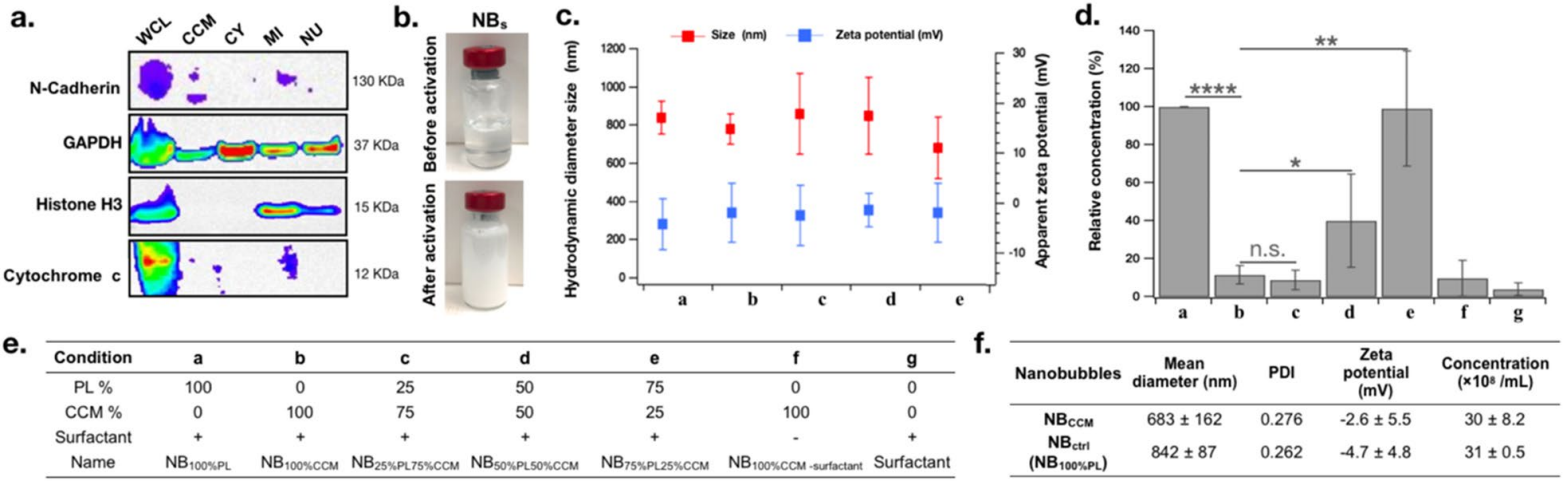
In vitro characterization of CCM-based NBs. **a** Western blot of different cellular fractions from MDA-MB-231 cells isolated by a differential centrifugation method for various cellular markers. The samples were probed using antibodies against GAPDH, Histone 3, Cytochrome C, and N-Cadherin. **b** Representative digital photographs of NBs before and after microfluidizer processing; **c** Particle zeta potential and size distribution measured using DLS; **d** relative concentration of NBs using single particle optical sensing. **e** Composition of each bubble tested in this study; (+) and (−) indicate the presence and absence of surfactant respectively in the formulation. **f** Characteristic summary of TNBC targeted NB_75%PL25%CCM_ (NB_CCM_) and non-targeted NBs (NB_ctrl_). WCL: whole cell lysate fraction; CY: cytoplasm fraction; MI: mitochondria fraction; NU: nucleus fraction; Data are shown as Mean ± SD. *P < 0.05,**P < 0.01, ****P < 0.0001

CCMs were isolated from TNBC MDA-MB-231 cells through cell lysis followed by differential centrifugations. Purity was confirmed by western blot using a series of intracellular and plasma membrane protein markers (Fig. 1a). The plasma membrane-specific marker, N-Cadherin, was present in the CCM purified fraction. Primarily located in the cytosol, GAPDH can also insert plasma membranes as an anchor protein. Accordingly, we observed some GAPDH signal in the CCM fraction. Conversely, there were traces of intracellular marker Cytochrome C in the CCM fraction, while nuclear Histone H3 was undetectable. For CCM-based NB formulation, we substituted various amounts of CCMs relative to commercial phospholipids (PLs), (0, 25, 50, 75 and 100 wt. %), supplemented with or without surfactant for surface tension reduction, and octafluoropropane gas. Based on the inherent self-assembly property of proteolipidic micelles, we used a microfluidizer device to establish a pressure-based disruption and reconstitution process for creation of biomimetic NBs. Following microfluidics processing, all compositions turned into stable milky emulsions (Fig. 1b). NB characterization for size and surface charge was realized using dynamic light scattering (DLS). No significant variation in zeta-potential was observed across the different conditions with a slightly negative average owing to the anionic nature of some PLs incorporated in the NB (Fig. 1c). Further particle size analysis revealed that all NBs were in the submicron range without significant difference related to CCM proportion. Importantly, the concentration of NB_100%CCM_ (condition 'b' in Fig. 1d) was significantly lower compared to non-targeted NBs formulated from synthetic PLs only (NB_100%PL_ also called NB_ctrl_ later in the text) (tenfold, *P* < 0.0001). However, introduction of synthetic PLs as part of the CCM-based NBs positively correlated with bubble concentration. NB_75%PL25%CCM_ (condition 'e' in Fig. 1d) exhibited the highest concentration among the CCM-based NBs, which was significantly increased compared to NB_100%CCM_ (tenfold, *P* = 0.0099), and similar to the one of non-targeted NBs. A summary table of each bubble composition is indicated in Fig. 1e. The mean diameter size and zeta-potential of NB_75%PL25%CCM_ were 683 ± 162 nm and − 2.6 ± 5.5 mV, respectively (Fig. 1f). In addition, NB_75%PL25%CCM_ showed good stability in an US phantom study, with similar kinetics as non-targeted NB (Additional file 1: Fig. S1).

### In vitro*,* TNBC cell surface proteins are conserved on CCM-based NBs

**Fig. 2:**
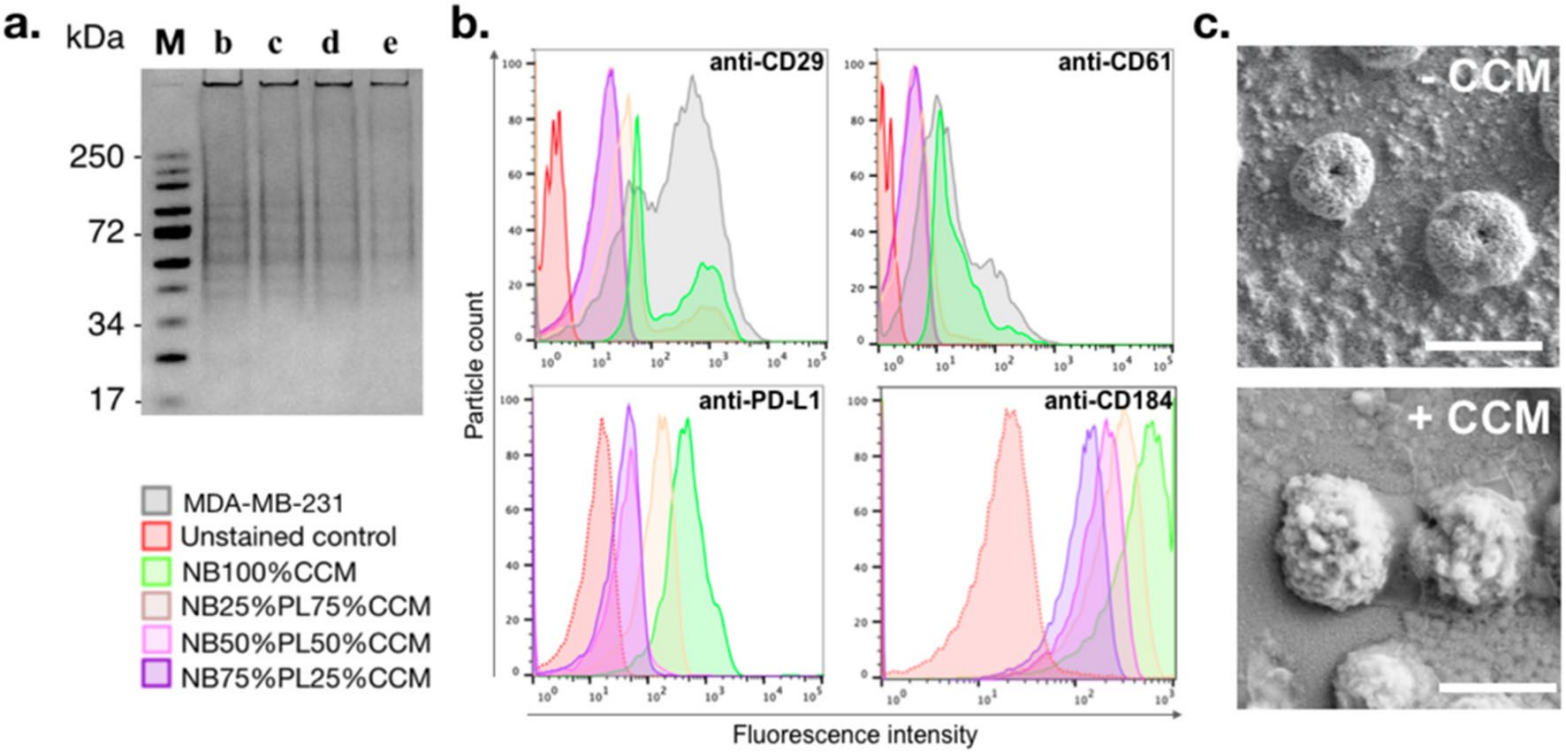
In vitro evaluation of the presence of CCM proteins on the shell of formulated NBs. **a** Coomassie blue stained proteins of NBs formulated with decreasing percentage of CCM resolved in 4–12% gradient SDS-PAGE (M: protein molecular weight marker; b-e refer to NB names as indicated in Fig. 1e); **b** FACS histogram showing the presence of CD29, CD61, PD-L1 and CD184 proteins on the surface of NB_CCM_; **c** SEM images showing the morphology of NB_ctrl_ (top) and NB_CCM_ (bottom). Scale bar = 1 μm.

With the aim of exploiting the ability of cancer cells for homotypic targeting, we analyzed the surfaceome conservation of MDA-MB-231 cells onto the different targeted NBs using in vitro assays (Fig. 2). The presence of CCM-associated proteins on the NBs from all compositions was first confirmed by gradient gel electrophoresis (Fig. 2a). We further analyzed the presence of CAMs, namely CD29 (integrin β1) and CD61 (integrin β3), as well as the programmed death-ligand 1 **(**PD-L1) and CD184 (CXCR4) by flow cytometry (Fig. 2b). Results confirmed that all types of CCM-based NBs presented detectable levels of surface proteins. Moreover, a morphological exam of NB_75%PL25%CCM_ and NB_100%PL_ was realized using scanning electron microscopy (SEM) (Fig. 2c). While non-targeted NBs showed a relatively smooth surface morphology, CCM enrichment induced pronounced morphological changes with rough and self-reconstituted membrane vesicles on the surface of the bubble shell, appearing as 50 nm to 100 nm structures. Based on our combined in vitro results, we decided to further test NB_75%PL25%CCM_ in our study, that is, the formulation that gave the highest bubble concentration while conserving tumor cell surfaceome. Henceforth, we abbreviated NB_75%PL25%CCM_ as NB_CCM_.

### MDA-MB-231-derived cell membranes target homologous TNBC cells in vitro

**Fig. 3:**
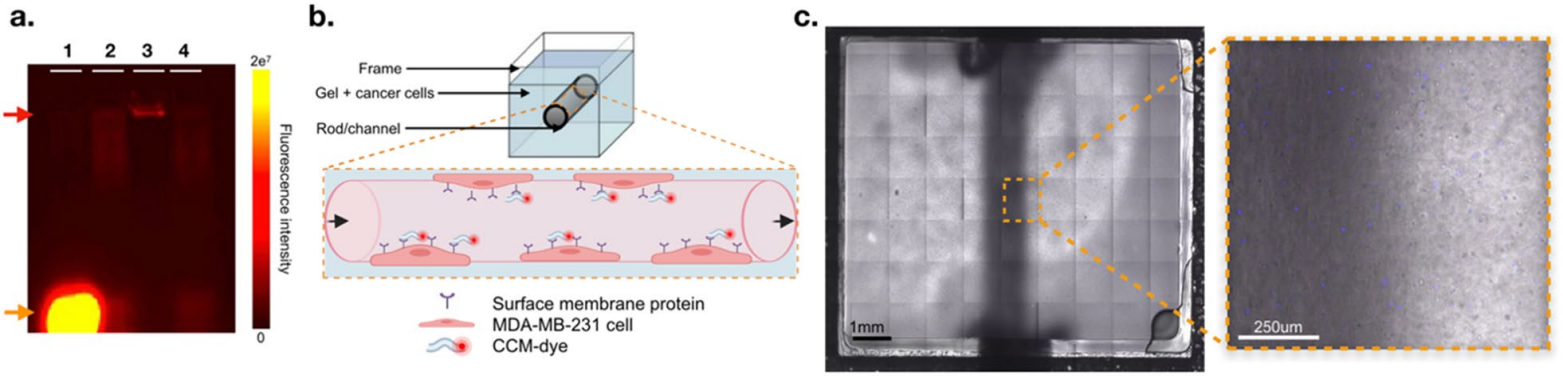
MDA-MB-231 cell-embedded phantom for CCM homotypic affinity assessment. **a** Purification of AF-647-conjugated CCMs (red arrow) from free dye (orange arrow) resolved in 2% agarose gel (1: free AF-647, 2: CCM before purification, 3: CCM purified pellet, 4: purified supernatant; **b** Phantom set-up; **c** Channel view under confocal microscopy with zoom-in portion showing Hoechst-stained cancer cells. Scale bar = 1 mm and 250 μm, respectively

**Fig. 4:**
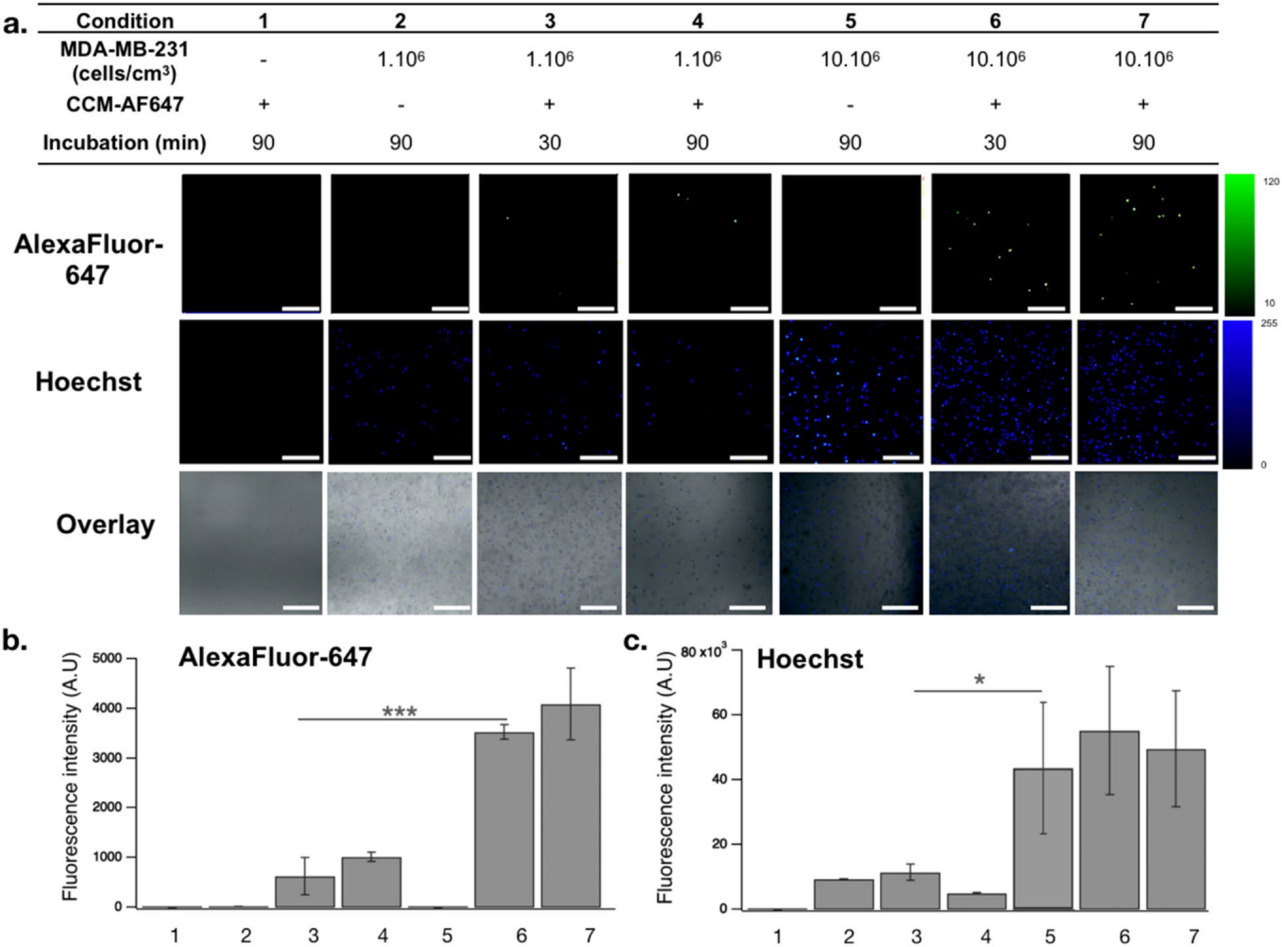
Homotypic affinity of AF-647-CCM towards parental MDA-MB-231 cells. **a** Bright-field and fluorescence imaging across different treatment conditions; **b** AF-647 fluorescence intensity; and **c** Hoechst signal intensity. Scale bar = 250 μm; Data are shown as Mean ± SD; *P < 0.05, ***P < 0.001

To confirm the CCM homotypic affinity property, we used a tissue-mimicking gel phantom containing MDA-MB-231 cells along a 2 mm inner diameter channel. AF-647-conjugated CCMs were injected into the channel for binding assays (Fig. 3). The channel was washed with PBS and the remaining fluorescence was analyzed by fluorescence imaging (Fig. 4a, b). Our results demonstrated that AF-647 fluorescence was retained only when both cancer cells and CCMs were present in the channel. Furthermore, cancer cell density positively correlated with AF-647 fluorescence intensity, confirming that higher exposure of cancer cell epitopes towards the channel allowed more interactions with CCMs. This supported the achievement of successful targeting of CCMs towards their parent cancer cells. In addition, the incubation times did not affect the homotypic affinity significantly, suggesting a relatively rapid and stable interaction of CCMs with their parent cells. Finally, we ensured that a similar cell density was used in each group by quantifying Hoechst 3342 signal intensity (Fig. 4c). Of note, such a phantom was made with US compatible materials thus allowing for further US flow and penetration assays, and optical imaging analysis on the same sample.

**Fig. 5:**
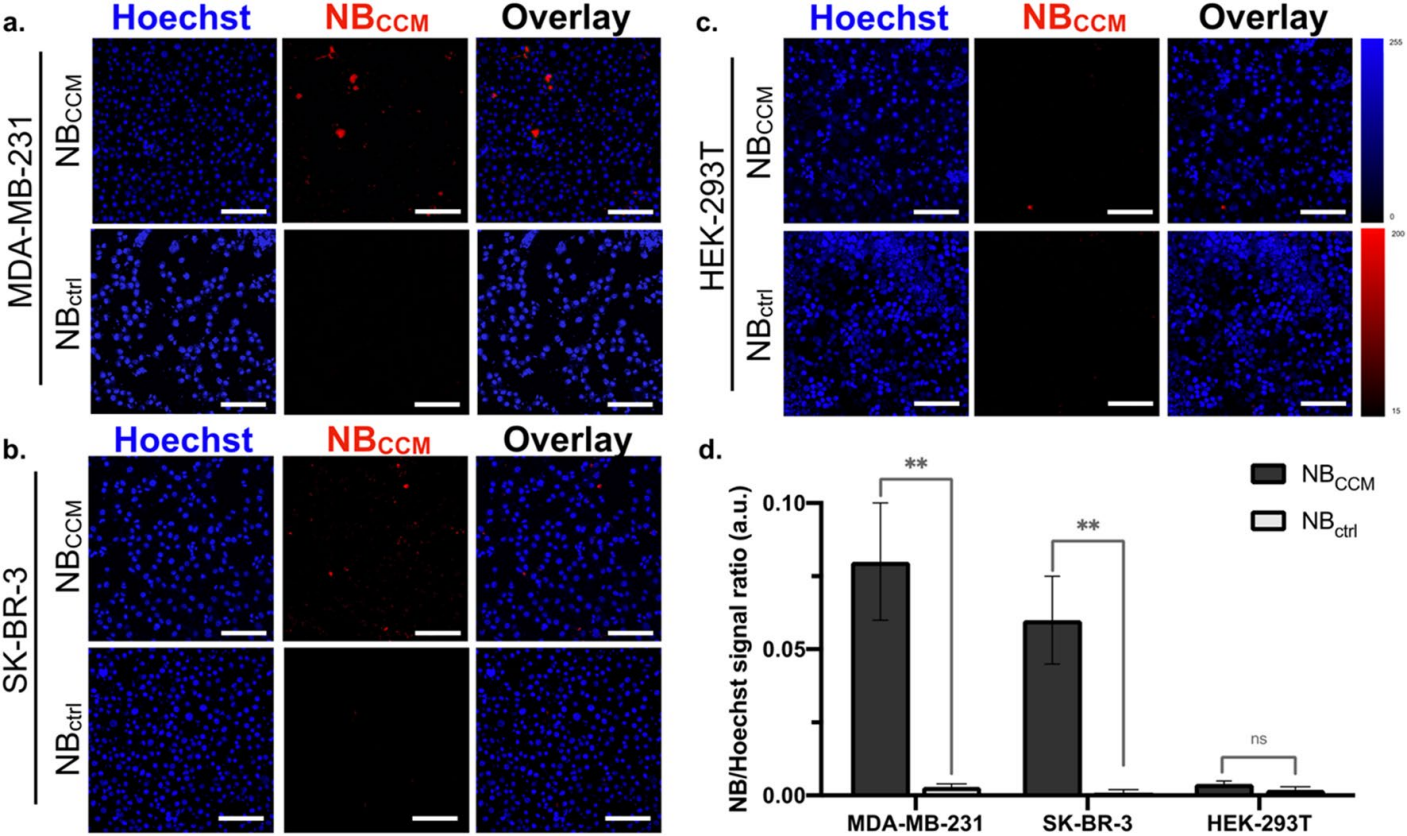
Affinity of MDA-MB-231-derived NB_CCM_ in various types of cells. **a** in MDA-MB-231 cells; **b** in SK-BR-3 cells; **c** in HEK-293T cells; **d** NB/Hoechst signal quantification. Scale bar = 100 μm; Data are shown as mean ± SD; **P < 0.01; (n = 3)

We further evaluated NB_CCM_ by studying homologous (with cancer cells) and heterologous (with non-cancerous cells) cell adhesion properties. For this purpose, we used phantoms containing MDA-MB-231 TNBC cells, HER2-positive SK-BR3 cells, or the immortalized non-neoplastic HEK-293T cells. After incubation with a fluorescently labeled NB_CCM_ or NB_ctrl_, we analyzed the fluorescence signal intensity using confocal laser-scanning microscopy (CLSM) (Fig. 5). NB_CCM_ signal intensity was significantly higher in phantoms containing MDA-MB-231 and SK-BR-3 cells compared to NB_ctrl_ (60-fold and 27-fold respectively, *P* = 0.002). In contrast, we did not observe any signal from the phantom integrated with HEK-293T cells (*P* = ns). The results of the fluorescence microscopic images revealed the specific adhesion of NB_CCM_ in homologous cancer cells. Interestingly, the co-incubation of NB_CCM_ with HEK-293T showed minimal fluorescence signal, likely owing to the lack of NB_CCM_ adhesion.

### NBCCM induces a higher US signal enhancement compared to NB_ctrl_ in a TNBC mouse model

**Fig. 6:**
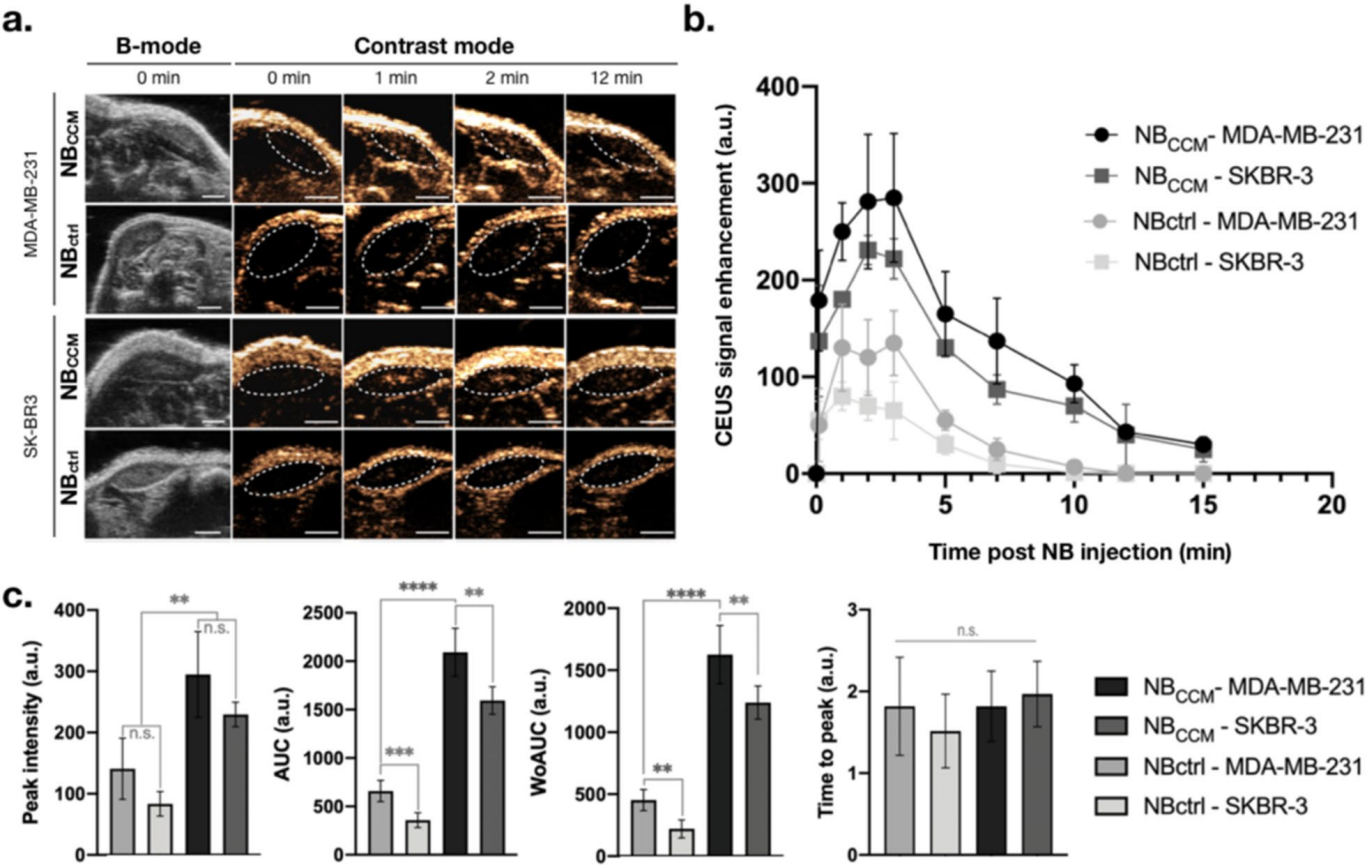
In vivo effect of NB_CCM_ and NB_ctrl_ on tumor enhancement by contrast-mode imaging. **a** Representative ultrasonographic images of SK-BR3 and MDA-MB-231 subcutaneous tumors before and after injection of NB_CCM_ or NB_ctrl_. The first and second columns show the B-mode and contrast-mode images of tumors before NB injection, respectively. The third to fifth columns show the non-linear signal at different time points after NB administration. Scale bar is 2 mm; **b** mean time intensity curves of both SK-BR3 and MDA-MB-231 tumors after NB injection; **c** comparison of NB kinetic parameters. Data are shown as Mean ± SEM; *P < 0.05, **P < 0.01, ***P < 0.001, ****P < 0.0001; (n = 5)

After thorough in vitro evaluation, the diagnostic capability of NB_CCM_ was evaluated in a dual-flank BC mouse model bearing MDA-MB-231 and SK-BR3 cells (n = 5 each). Tumors were localized using US in B-mode imaging. When tumor size reached about 70 mm^3^, contrast mode imaging was used to specifically detect nonlinear signals (Fig. 6a). After tail vein bolus sequential injections of NB_ctrl_ and NB_CCM_ (200 μL, 6 × 10^8^ NB/mL), US contrast images were acquired regularly to determine the perfusion and bubble dispersion extracted from the time-intensity curves (TIC) (Fig. 6b). NB kinetic parameters (*i.e.,* time to peak, peak intensity, area under the curve (AUC), and area of wash-out (WoAUC)) in each tumor were compared between both bubble types (Fig. 6c). We observed a rapid signal enhancement in both tumors and for both NB types approximately 1 min to 2 min after injection. The times to peak were not significantly different between all conditions, signifying a similar dynamic of NB_CCM_ and NB_ctrl_ in the bloodstream. The average peak intensity was significantly increased for NB_CCM_ compared to NB_ctrl_ in MDA-MB-231 and SK-BR3 tumors (2.1-fold, *P* = 0.004; and 3.5-fold, *P* = 0.006, respectively), indicating a higher stability of NB_CCM._ Furthermore, there was no significant difference between the peak intensity for NB_CCM_ in either MDA-MB-231 or SK-BR3 tumors (*P* = 0.1942) indicating comparable tumor morphology and vasculature. The total area under the curve was significantly increased with NB_CCM_ compared to NB_ctrl_. Importantly, we calculated a separate AUC during the wash-out phase, where the main differences in bubble dynamics were expected owing to tumor retention. The WoAUC of NB_CCM_ was significantly increased compared to NB_ctrl_ in both MDA-MB-231 (3.6-fold, *P* < 0.0001) and SK-BR3 tumors (7.4-fold, *P* < 0.0001). Moreover, WoAUC of NB_CCM_ in MDA-MB-231 tumor was 1.3-fold higher than in SK-BR3 tumor (*P* = 0.0125). However, WoAUC of NB_ctrl_ between the two tumors was also significantly different (twofold, *P* < 0.0016) indicating a different EPR effect on each BC subtypes. Therefore, such differences could have also affected NB_CCM,_ and it is likely that the WoAUC were similar between the two tumors. Consistent with our previous in vitro results, similar affinity of NB_CCM_ on different BC subtypes could be owing to a common expression of CAMs. Overall, these results indicate a higher stability and prolonged retention of NB_CCM_ in TNBC tumors.

### NBCCM extravasates from blood vessels and targets tumor cells

**Fig. 7:**
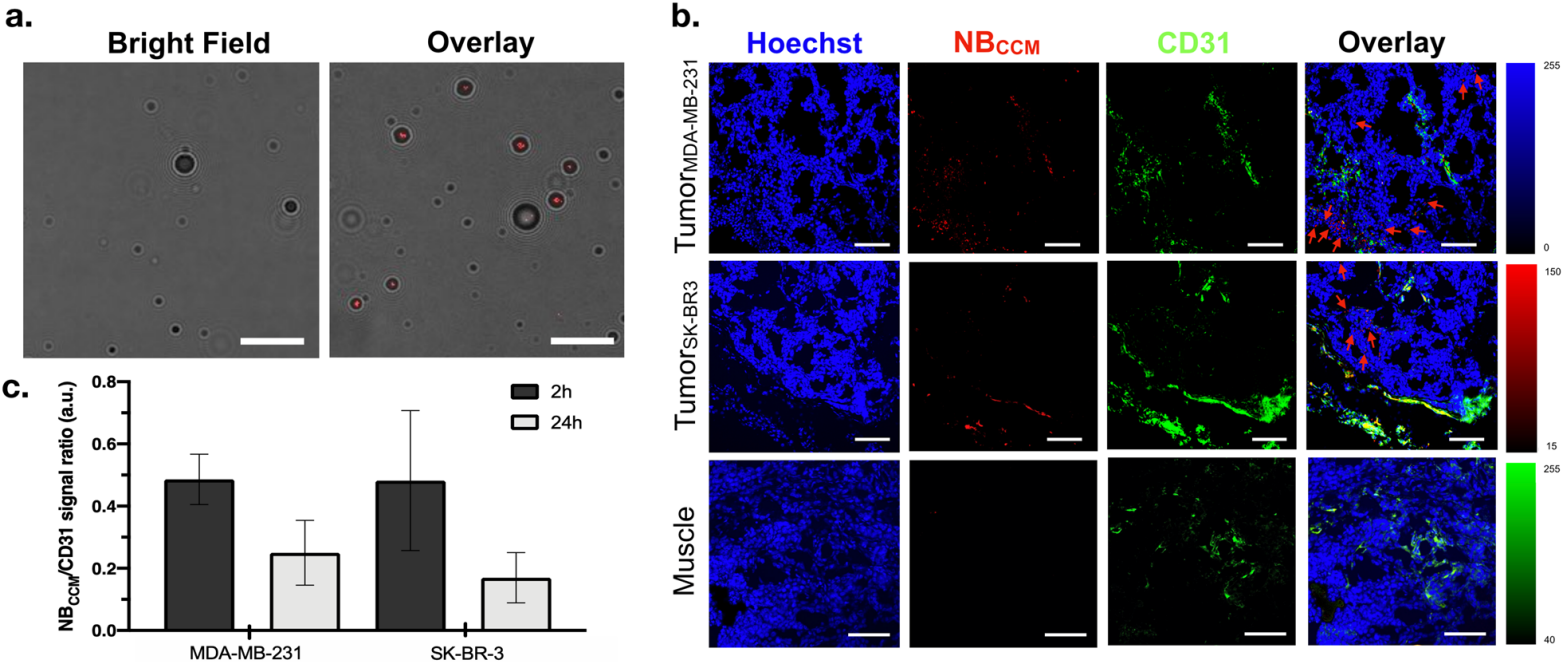
Fluorescence images of ICG-NB_CCM_. **a** In vitro fluorescence images of NB_CCM_ under microscope at ×400 magnifications with the corresponding bright field image. Scale bar = 10 µm. **b** Representative CLSM images of frozen tumor and skeletal muscle sections after vessel (CD31) and nucleus (Hoechst 33342) labeling with corresponding bright field images. Red arrows indicate principal positions of extravascular NB_CCMs_. Scale bar = 100 µm; **c** quantification of fluorescence ratio in both tumors (total bubbles fluorescence/vessels fluorescence). Data are shown as mean ± SD

After in vivo contrast imaging, we prepared NBs using indocyanine green (ICG) dye conjugated CCM (i.e., ICG-NB_CCM_) to assess their extravasation and localization within tumors using CLSM. In vitro, fluorescence images showed the presence of ICG signal in NB_CCM,_ observed as a uniform red signal co-localized with the corresponding bright field image (Fig. 7a).

In addition, we demonstrated the specific penetration of NB_CCM_ into tumors in a new set of animals bearing subcutaneous MDA-MB-231 and SK-BR3 tumors on each flank. We injected NB_CCM_ via tail vein and animals were sacrificed 2 h or 24 h after injection (n = 3 each group). No US imaging was performed in these animals. Major organs (lungs, heart, liver, spleen, kidneys), both tumors, and adjacent skeletal muscles were harvested for histological analysis using CLSM. Both tumor and muscle sections were stained using anti-CD31 antibody as a vascular marker. After 2 h, NB_CCM_ was abundantly present in the vasculature of both SK-BR3 and MDA-MB-231 tumors (Fig. 7b). Importantly, NB_CCM_ signal was detected deep into the tumors (as far as approximately 125 µm distant from the vasculature), providing robust evidence of extravasation of NB_CCM_ from the vasculature and entering the tumor microenvironment (Additional file 1: Fig. S3). In addition, there were no significant differences between the two BC subtypes in NB_CCM_ retention (Fig. 7c), consistent with our US imaging findings. Interestingly, animals sacrificed 24 h after NB_CCM_ injection showed the presence of ICG fluorescence in both tumors, consistent with a specific and prolonged retention of NB_CCM_ in the tumor (Fig. 7c, Additional file 1: Fig. S2). Conversely, we did not detect NB_CCM_ in the vessels or extravascular spaces of skeletal muscle sections at any time point. Moreover, we further assessed NB_CCM_ distribution in the lungs, heart, liver, muscle, kidneys, and spleen of each treated animal by direct analysis of ICG signal intensity (Additional file 1: Figs. S4, S5). Our results showed no evidence of NB_CCM_ accumulation in the heart, kidneys, and muscle at both the time points. We observed traces of NB_CCM_ in the liver and the spleen 2 h post-injection (5–13% compared to the mean signal intensity in the tumors), with undetectable signals 24 h post-injection (Additional file 1: Fig. S5). Besides the tumors, the lungs retained a relatively high amount of NB_CCM_ (40%) at 2 h post-injection yet was rapidly cleared with no signal remaining after 24 h.

### Histological analysis

**Fig. 8:**
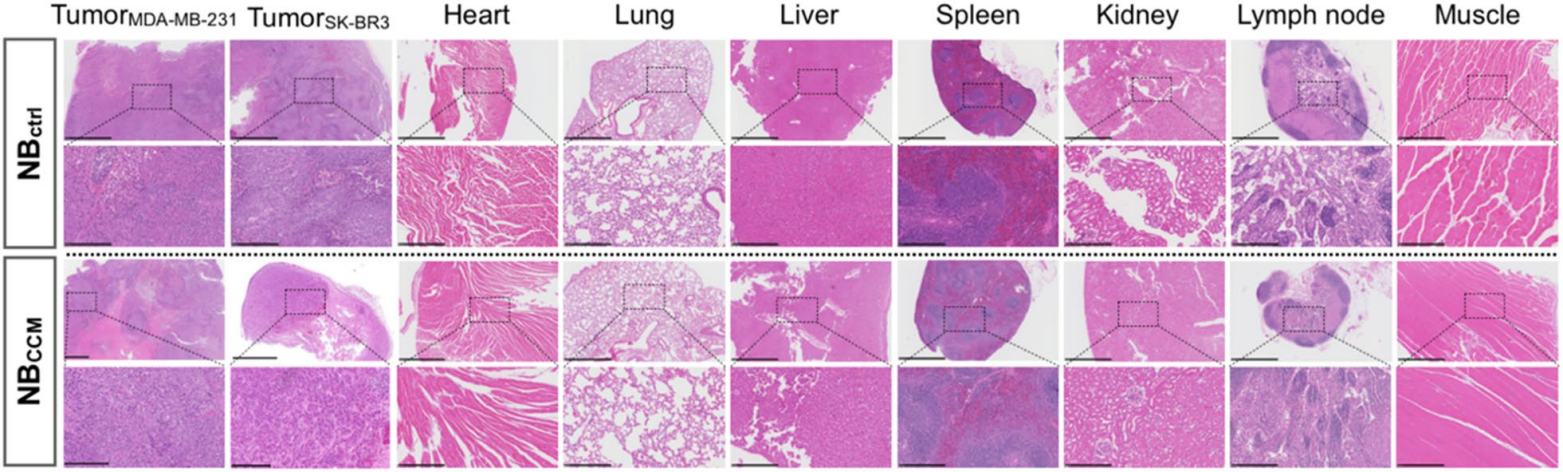
Representative H&E images of mouse tissues. The first and third lanes show heart, lung, tumors, kidney, liver, spleen, lymph node, and muscle sections treated by NB_ctrl_ or NB_CCM,_ respectively. Scale bar = 1 mm. The second and fourth lanes show corresponding magnified areas. Scale bar = 100 µm

Representative H&E-stained sections of major organs (heart, liver, spleen, lungs, and kidneys), tumor tissues and skeletal muscle are shown in Fig. 8. We found no evidence of structural abnormalities in different organs of mice treated using either NB_ctrl_ or NB_CCM_. Overall, the NB injection and CEUS method did not cause any tissue damage.

## Discussion

BC is the most frequently diagnosed cancer and the second leading cause of cancer mortality in women worldwide [28]. Lacking the expression of ER, PR, and HER2, TNBC is a heterogeneous BC subtype with the most aggressive clinical course, and its early detection is key to improving survival. Although mammography remains the main modality for BC screening, accuracy of diagnosis is limited in dense or heterogenous breast tissues. Thus, standard US is often performed as complementary test to assess suspicious lesions, but frequently results in overdiagnosis, with low positive predictive value of 5.6–8.6% [29, 30]. Beyond anatomical imaging, CEUS using contrast agents targeting specific oncogenic markers holds potential for accurate early detection. Although first targeted MBs have reached clinical trials (e.g., KDR-targeted MB (BR55)), there is still considerable room for improvement [9]. Recently, we and others have developed a new tumor vascular B7-H3 targeted-MBs for BC diagnosis [31, 32]. However, MBs have a short in vivo half-life (typically < 5 min) [24], and are restricted to vascular targets only. Conversely, because of their capacity to extravasate beyond the blood vessels to target biomarkers on tumor cells directly, NBs have broadened the choice of targets and opened the possibility for increased specificity and detection sensitivity. Furthermore, studies have demonstrated that NBs with phospholipid shells and gas cores had longer tumor specific accumulation compared to clinical MBs in vivo owing to the EPR effect [14, 33, 34]. To date, cancer targeted NBs are formulated either by conjugation of a cancer specific ligand onto the shell of a preformed bubble, or the cancer specific ligand is conjugated to one constituent element of the shell before mixing all the components for bubble formulation. However, lacking conventional BC markers, TNBCs do not respond to targeted NB strategies that have shown success in HER2-positive subtypes [12]. The goal of this study was to formulate the first CCM-based targeted NB (NB_CCM_) by exploiting the homotypic affinity of cell membranes to achieve specific US molecular imaging of TNBC in mice.

Recently, we developed a microfluidic technology using a pressure-based disruption and reconstitution process as a means to produce biomimetic nanovesicles [18, 35]. This method takes advantage of the self-assembly property of cell membranes in aqueous solutions as proteolipid micelles to create hybrid biomimetic membranes. Microfluidics allows the production of millions of particles in short timeframes, with excellent control over cell size and cargo, and has been used for artificial cell synthesis [36, 37]. This process is also clinically scalable and provides an independent control over particle chemical composition. Here, we optimized CCM concentrations, and the formulation needed for stable biomimetic NBs. The average hydrodynamic diameters of NB_CCM_ and NB_ctrl_ that we formulated were 683 ± 162 nm and 842 ± 87 nm, respectively. The negative charge of anionic DSPE-PEG (5000) and DPPA helps prevent NB aggregation, and the hydrophilic character can also keep the NB water dispersible. SEM images provided direct visualization of NB size and surface morphology. Analysis of several surface protein expressions confirmed the conservations of integrins CD29 and CD61, and oncogenic therapeutic targets such as PD-L1 and CD184. Microfluidics could allow for enrichment with recombinant CAMs known to facilitate homotypic recognition, at the right density that matches the cancer heterogeneity of a particular patient. Moreover, the specific homotypic affinity of MDA-MB-231-derived CCM and NB_CCM_ was confirmed in vitro. Our findings were consistent with previously published studies assessing the homotypic targeting property of 4T1-cell membrane [17]. The stability of NB_CCM_ and NB_ctrl_ under US imaging conditions exceeded 3 h after formulation, with similar in vitro CEUS enhancement capability. We performed in vivo acoustic evaluation of NB_CCM_ in mice implanted with HER2-positive BC cells (SK-BR3) and TNBC cells (MDA-MB-231), and NB_CCM_ was used to image a dual-flank BC model. Significant differences were observed in peak intensity, AUC and WoAUC between NB_CCM_ compared to non-targeted NBs of both BC subtypes. WoAUC was significantly enhanced with NB_CCM_ compared to NB_ctrl_ in MDA-MB-231 tumors and tended to be higher than in SK-BR3 tumors, indicating prolonged retention of NB_CCM_ in TNBC. In addition, our group identified several adhesion proteins that could participate in homologous recognition of cancer cells [17]. Thus, overlap in CAM expression between these two BC subtypes is likely to give similar recognition by NB_CCM_ as we demonstrated in both in vitro and in vivo studies. Mass spectrometry analysis comparing the plasma membrane proteomes of various BC cell lines has shown 62% shared proteins between SK-BR3 and MDA-MB-231 cell lines [38]. Interestingly, we and others have previously shown that bubbles are primarily captured by Kupffer cells in the liver for phagocytosis and by pulmonary and splenic macrophages and mononuclear phagocytes as part of their clearance mechanism in human [39, 40]. Our biodistribution study was consistent with the literature. Immunofluorescence analysis of ICG-NB_CCM_ demonstrated that NB_CCM_ was absent from healthy tissues where endothelial cells are tightly sealed, further supported by pericytes, making endothelial gaps typically less than 7 nm [41]. Conversely, the incomplete endothelial lining of tumors creates large entryways (from 100 to 1000 nm) [42] for ICG-NB_CCM_ that accumulated in both tumors distant from the vasculature. No evidence of cellular damage caused by NBs CEUS was observed in any major organ.

We acknowledge that NB_CCM_ extravasation could be improved by reducing the mean diameter of the bubbles after performing slow differential centrifugations on the formulated bubbles to isolate the relevant subpopulation [43]. However, lower bubble sizes will require higher US frequencies that are not clinically applicable. Moreover, while the EPR effect is widely believed to improve passive tumor targeting, limiting factors include the heterogenous blood supply within a tumor, high interstitial fluid pressure, growth-induced solid stress, and solid stress from abnormal matrices [44]. In addition, NB properties (i.e., size, shape, surface charge, compressibility, stiffness, porosity, composition and targeting ligands) can also influence such biological processes, altogether affecting the effectiveness of the EPR effect. Therefore, by adding an active tumor targeting as well as locally delivered mechanical forces, we aimed to potentiate the accumulation of NBs in the tumor microenvironment, thus overpassing the solely passive EPR effect for future therapeutic applications. Specifically, sonoporation, a process using ultrasonically activated UCAs, is known to locally and momentarily enhance cell membrane or endothelial layer permeability by forming pores up to several microns, increasing extravasation efficiency of various compounds [45, 46].

## Conclusion

Compared to conventional targeted UCA formulations, our biomimetic bubbles allow for personalized medicine that could offer the best chances for successful TNBC targeting. We anticipate that the cell membrane-based NBs presented in this work are only a starting point for future engineering of targeted UCAs that have the potential for high impact in oncology. This approach could also be used in immunomodulatory therapies by serving as cancer vaccines, for targeted drug delivery purposes, and in many more diagnostic and therapeutic applications.

## Materials and methods

### Cell culture

Human MDA-MB-231 TNBC cells, HER2^+^ SK-BR3 cells, and HEK-293T (ATCC HTB-26, HTB-30, and CRL-3216 respectively) were cultured in Dulbecco’s Modified Eagle Medium (DMEM) supplemented with 10% fetal bovine serum (FBS), 100 U/mL penicillin and 0.1% streptomycin (all from ThermoFisher Scientific, USA), and maintained in a 37 °C incubator with 5% CO_2_ and 95% air.

### MDA-MB-231 cell membrane isolation and immunoblots

MDA-MB-231 cells were grown to full confluency for the isolation of CCMs. Cells were detached from the plates using 0.25% trypsin–EDTA (Invitrogen, Carlsbad, CA) and washed in PBS three times by centrifuging at 500×*g* for 3 min. The cell pellet was suspended in ice cold hypotonic buffer (10 mM Tris–HCl, 2 mM MgCl_2_, pH = 7.3 (Sigma Aldrich), with 1 mM PMSF. After 5 min of incubation, NP10 cell lysis buffer was added to the cell suspension (7.7% v/v) and incubated further on ice for 30 min. The solution was gently vortexed once every 10 min and cell membrane disruption was visually controlled under microscopy before centrifugation at 500×*g* for 5 min at 4 °C. The supernatant was saved and subjected to a second centrifugation at 20,000×*g* for 10 min. The supernatant was collected and subjected to centrifugation at 100,000×*g* for 30 min to collect the CCM in the pellet. The pellet containing the CCM was then washed once in PBS and used for further evaluation and NB formulations. The various fractions were resolved in 4–12% gradient SDS-PAGE gels (25 µg/lane) and electroblotted onto a 0.2 µm pore size nitrocellulose membrane (Bio Rad, Hercules, CA) for immunoblot analysis using different cell markers. Membranes were blocked in PBS containing 0.05% Tween-20 (PBS-T) with 5% non-fat dry milk powder for 30 min and detected independently using GAPDH, Histone H3, N-Cadherin, and Cytochrome C primary antibodies at the recommended dilutions (Cell Signaling Technology, Danvers, MA) by incubating overnight at 4 °C on a shaker. Membranes were then incubated with HRP-conjugated IgG secondary antibody. After a washing step, signals were developed with the addition of enhanced-chemiluminescence (ECL) substrate (Thermo Fisher Scientific, USA) and imaged using IVIS Lumina III *In-Vivo* Imaging System (Perkin Elmer, Santa Clara, CA).

### Preparation of fluorescent dye conjugated CCM for in vitro and in vivo studies

CCMs were conjugated to either AF-647 or ICG fluorescent dyes (Thermo Fisher Scientific, USA) using N-Hydroxysuccinimide (NHS) ester chemistry by incubating for 12 h at 4 °C for further applications in vitro and in vivo. Dye-labelled CCMs were purified by centrifuging at 100,000×*g* for 30 min, and the unconjugated dye was removed. Successful conjugation was checked by resolving fractions in 2% agarose gel electrophoresis. ICG-NB_CCM_ was visualized directly in vitro and ex vivo using CLSM.

### Synthesis of NBCCM and NB_ctrl_

We used commercial PLs such as DPPA (1,2-dipalmitoyl-*sn*-glycero-3-phosphate), DPPC (1,2-dipalmitoyl-*sn*-glycero-3-phosphocholine), DSPE-PEG-5000 ((1,2-distearoyl-*sn*-glycero-3-phosphoethanolamine-*N*-methoxypolyethylene glycol)-5000) (Avanti Polar Lipids, Inc., Alabaster, AL) dissolved in sterile saline at the molar ratio of 7:55:5 (0.75 mg total of lipid constituents) for the formulation of NBs. PLs and CCMs, solubilized and homogenized separately using a LV1-microfluidic system (Microfluidics, Westwood, MA), were used for NB preparations. LV1-microfluidic system was set at 30,000 psi, its working track was washed five times with 75% ethanol solution, then re-washed three times with saline solution to prepare the various formulations. The PL mix or CCMs were loaded into the microfluidic system, and the solubilized solution was extracted at the outlet. We performed this process three times for complete solubilization. Next, we added the CCM solution with the adequate percentages relative to the commercial PL mix (i.e., 100%, 75%, 50%, 25% and 0% in weight, 1 mg/mL stock) for various formulations. To prepare the tumor-targeted NBs (NB_CCM_) and non-targeted NBs (NB_ctrl_), each solution was supplemented with a non-ionic copolymer surfactant, Pluronic F-127 (0.03 mg/mL; Sigma-Aldrich, St. Louis, MO), glycerol (125 mg/mL; Sigma-Aldrich, St. Louis, MO) and propylene glycol (105 mg/mL; BioWorld, Dublin, OH) into 3 mL glass vials (Wheaton, Millville, NJ). Vials were sealed before filling with octafluoropropane (C_3_F_8_) gas (Fluoromed, L.P., Round Rock, TX). Vials were agitated using a Vialmix (Lantheus Medical Imaging, Inc., North Billerica, MA) for 45 s. The MBs were processed using a microfluidizer by running at 30,000 psi to obtain homogenous NB populations.

### Dynamic light scattering (DLS) for size and charge measurement, and particle counting by accusizer optical sizing device

The mean hydrodynamic diameters and zeta-potential (surface charge) of the NBs were measured using dynamic light scattering (DLS) with a scattering angle of 90° (Zetasizer Nano ZS90 sizing device, Malvern Panalytical Ltd., Malvern, U.K.). Samples were dispersed in distilled water at the required dilution for DLS measurement. We averaged the readings collected from three measurements to obtain the results. The concentration of NBs was determined using a single particle optical sizing device (0.5 to 400 μm measurable range, Accusizer 770A, Particle Sizing Systems, Santa Barbara, CA, USA).

### Surface analysis of NBs bearing CCMs

Cell membrane protein content was analyzed for the different NB constructs on 4–12% gradient SDS-PAGE (30 µg/lane) followed by Coomassie Blue staining (SimplyBlue SafeStain, Carlsbad, CA) for visualization using a BioRad Gel-Doc system. CCM proteins were further investigated by FACS. Bubbles were incubated with various human reactive antibodies CD29-APC, CD61-PE, PD-L1-FITC, and CD184-CXCR4-APC (all were from BioLegend, San Diego, CA) at the recommended dilutions for 10 min at 25 °C. After washing once with PBS, fluorescence intensity from the bubbles was measured using a flow cytometer (Guava easyCyte; Luminex Corp., Austin, TX) and quantified using FlowJo software (Tree Star, Ashland, OR, USA).

### Morphology characterization of NBCCM using SEM

Scanning electron microscope (SEM, Zeiss Sigma) was used to assess the NB_CCM_ and NB_ctrl_ surface morphology and architecture. Images were collected at 3000× magnifications with an accelerating voltage of 2.0 kV.

### CLSM imaging of CCM targeting properties in a gel phantom

To study the homologous and heterologous adhesion property of CCMs, a custom 3D cancer tissue mimicking phantom was prepared. The phantom body was formed within a plastic frame with rods placed horizontally to mold flow channels. Gel dimensions were 0.9*0.8*1.4 cm. Each phantom contained one straight channel of 1.2 mm diameter, with fittings on the frame for connection to a syringe pump and drain tubing. Gelatin (5.55% w/w) and agar (2.02% w/w) were mixed in water at 80 °C under high-speed stirring to ensure a homogeneous solution. Gels were cooled to 37 °C, and supplement with Hoechst 33,342 (Invitrogen, Carlsbad, CA) stained MDA-MB-231, SK-BR3, or the immortalized non-neoplastic HEK-293T cells (1–10 × 10^6^). Molds were filled immediately after and stored at 4 °C for 4 h. When the gel solidified, the rods were manually removed, and the flow channels were cleaned using water. Dye-labeled CCM was injected (4.9 mg/mL) in the channels and incubated for 30 or 90 min at 25 °C after which the channels were washed 5 times with water. In addition, dye labeled CCM was used to formulate fluorescently labeled NBCCM (same procedure as for NBCCM) to assess the targeting properties of the biomimetic bubble itself in the same phantoms. ICG-NBCCM (100 μL) were used to fill the channels and incubated for 2 h. Images were acquired using a Leica TCS SP8 laser confocal microscope.

### Murine subcutaneous breast tumor models

Xenograft mouse models of human BCs were used. MDA-MB-231 and SK-BR3 cells were cultured to 70–80% confluency, trypsinized, counted in a hemocytometer, and resuspended in 50 µL PBS mixed with 50 µL of Matrigel (BD Biosciences, San Jose, CA) for implantation. MDA-MB-231 and SK-BR3 cells were implanted (10^6^ cell/site) in the lower left and right flanks of female nude (NU/NU) mice (6 weeks old, Charles River, Wilmington, MA) and allowed to grow for 11 to 13 days. Tumor growth was assessed daily using caliper measurements in two dimensions and US imaging one day prior to utilization. When tumor volumes were approximately 70 mm^3^ (Volume = 0.5 × (width)^2^ × (length)), animals were used for imaging studies.

### In vivo US molecular imaging experimental design

We used nude mice bearing both MDA-MB-231 and SK-BR3 tumors for US molecular imaging using two NB constructs (NB_CCM_ and NB_ctrl_). Animals were anesthetized using 1–2% isoflurane in 0.5–1 L/min oxygen. A total of 6 × 10^8^ NBs (200 μL) was sequentially injected by intravenous bolus injection via tail vein to acquire contrast enhanced ultrasound images. All in vivo imaging studies were performed in contrast mode using a dedicated small animal high resolution US imaging system with the transducer placed over the tumor, guided by B-mode imaging to detect the target tissue of interest. Contrast mode images were acquired using an 18 MHz linear transducer (MS250), with the following imaging parameters: focal length, 10 mm; transmit power, 4%; mechanical index, 0.2; and dynamic range, 40 dB. These parameters were kept constant in all imaging sessions. A waiting interval of minimum 1 h was maintained between each NB injection to allow for complete clearance before the next imaging. Any remaining attached NBs were destroyed by applying a high-power destruction pulse (1 s continuous high-power destructive pulse of 3.7 MPa, transmit power, 100%; and mechanical index, 0.63).

### Ultrasound molecular imaging data analysis

Molecular imaging signals were quantified post image acquisitions with correction for breathing motion artifacts using Vevo 2100 integrated analysis software (VevoCQ; VisualSonics). Regions of interest (ROIs) were manually drawn outlining tumor areas for contrast signal quantification. The magnitude of non-linear signal from NBs was measured as a function of time (time-intensity curve—TIC) and expressed in arbitrary units (a.u.). Bubble kinetic parameters were analyzed using PRISM v6.0 (GraphPad).

### Hematoxylin and eosin staining

At the end of imaging studies, tumors were excised together with major organs and tissues of interest, and fixed in 4% paraformaldehyde (Santa Cruz Biotechnology Inc., CA) at 4 °C for 24 h, then immersed in 70% ethanol before using for routine hematoxylin and eosin staining (Stanford Animal Histology Services). Slides were imaged using a Nanozoomer (Hamamatsu, Japan) and digitally evaluated for any architectural changes.

### Ex vivo localization of NBCCM after immunofluorescence staining

To confirm that NB_CCM_ were small enough to pass through the endothelial gaps in tumors, we used CLSM of tissues to determine the location of ICG-NB_CCM_ ex vivo. Tumor-bearing mice were randomly separated into two groups and bolus injected ICG-NB_CCM_ (6 × 10^8^ NBs in 200 μL) via tail vein. After 2 h or 24 h post injection, animals were sacrificed and major organs (lungs, heart, liver, spleen, kidney), skeletal muscle and tumors were collected, fixed in 4% paraformaldehyde, embedded in optimal cutting temperature (OCT) medium (Sakura Finetek, Torrance, CA), and placed in a -80 °C freezer. The tissues were then cryosectioned into 10 μm slices using a cryomicrotome (Leica CM1850, Wetzlar, Germany). The cryostat temperature was set between − 15 °C  − 24 °C and adjusted to achieve an isothermal state for each sample. Slides were air dry for 30 min at room temperature to adhere tissues strongly onto the slides, before washing with PBS. Tumors and muscle sections were incubated in a blocking solution of 2% bovine serum albumin and 1% normal donkey serum (both from Sigma, St. Louis, MO, USA) for 60 min at room temperature in a humidifying chamber. The slices were stained for vascular endothelial cell marker CD31 (Anti-CD31-AF488, 1.5 μg/test, BioLegend, San Diego, CA) diluted in incubation buffer at 4 °C overnight. All tissue slides were then washed in PBS and supplemented with 100 µL of a Hoechst solution for 5 min at room temperature. Excess of dye was removed by PBS and water washes. Slides were mounted using an anti-fade mounting media (Vector Laboratories, Burlingame, CA) for confocal fluorescence microscopy visualization. We imaged tissue slices using a Leica TCS SP8 laser confocal microscope. Images of the ICG-NB_CCM_, vessels, and nuclei were captured in separate color channels.

### Statistical analysis

Statistical analysis was performed using PRISM v6.0 (GraphPad). All results are presented as mean ± standard deviation (S.D.) unless specified otherwise. Student-t test was applied to determine statistical significance between groups.

## Supplementary Information

**Additional file 1. Supplementary Materials and Methods. **CEUS imaging of NBCCM and NBctrl in a gel phantom. **Supplementary Results and Discussion. **NBCCM exhibits high stability under CEUS imaging in vitro. **Supplementary Figures. **Figures S1 to S5.

## Data Availability

All data generated or analyzed during this study are included in the article and additional file.

## Abbreviations

WoAUC: Area of wash-out
AUC: Area under the curve
BC: Breast cancer
CA-125: Cancer antigen 125
CCM: Cancer cell membrane
CAM: Cell adhesion molecules
CLSM: Confocal laser-scanning microscopy
CEUS: Contrast-enhanced US
DLS: Dynamic light scattering
EPR: Enhanced permeability and retention
ER: Estrogen receptor
HER2: Human epidermal growth factor 2-receptor
ICG: Indocyanine green
MRI: Magnetic resonance imaging
MB: Microbubbles
NB: Nanobubble
PL: Phospholipids
PR: Progesterone receptor
PSMA: Prostate specific membrane antigen
SEM: Scanning electron microscopy
TIC: Time-intensity curves
TNBC: Triple-negative breast cancer
US: Ultrasound
UCA: Ultrasound contrast agent
