# Supplementary material for: Biomimetic nanobubbles for triple-negative breast cancer targeted ultrasound molecular imaging
Source: J Nanobiotechnology. 2022 Jun 10;20:267. doi: 10.1186/s12951-022-01484-9 (PMC9185914; doi:10.1186/s12951-022-01484-9)
Supplement: Supplementary file 1 — Additional file 1. Supplementary Materials and Methods. CEUS imaging of NBCCM and NBctrl in a gel phantom. Supplementary Results and Discussion. NBCCM exhibits high stability under CEUS imaging in vitro. Supplementary Figures. Figures S1 to S5. [file 12951_2022_1484_MOESM1_ESM.pdf]

Supplementary Information

Biomimetic Nanobubbles for Triple Negative Breast Cancer Targeted Ultrasound Molecular Imaging

Natacha Jugniot[1,2], Tarik F. Massoud[1], Jeremy J. Dahl[2]

and Ramasamy Paulmurugan[1,2*]

[1]Molecular Imaging Program at Stanford (MIPS), and Bio-X Program, Department of Radiology, School of Medicine, Stanford University, Stanford, California - 94305-5427 USA

[2]Canary Center at Stanford for Cancer Early Detection, Department of Radiology, School of Medicine, Stanford University, California - 94305-5427 USA

Supplementary Materials and Methods

CEUS imaging of NB_{CCM} and NB_{ctrl} in a gel phantom

To analyze our NB stability, a gel phantom was used for US imaging (Vevo 2100, FUJIFILM VisualSonics, Inc., Toronto, ON, Canada). A phantom was created to fit the US probe field of view (FOV). The gel dimensions were 10*7*1.5 cm with a 2 mm diameter channel for imaging. We applied the same procedure described in the «**CLSM imaging of CCM homotypic targeting in a gel phantom**» section, except that there were no cancer cells for imaging the blank channel. The gel surface was covered with water, and the US transducer (MS250, VisualSonics; lateral and axial resolution of 165 µm and 75 µm, respectively) was placed in contact with the phantom surface for clean visualization of the channel. NB_{CCM} were infused in the tubing (4.5 mL, 1.10^8 particle/mL) and continuously circulated the bubbles using a peristaltic pump (flow rate = 0.2 mL/min). We recorded the non-linear signal for 180 min after which a high-pressure destructive pulse was applied (1-second continuous high-power destructive pulse; mechanical index, 0.63) to destroy all the bubbles in the FOV. We analyzed the non-linear signal replenishment using a Vevo 2100 integrated analysis software (VevoCQ; VisualSonics).

Supplementary Results and Discussion

NB_{CCM} exhibits high stability under CEUS imaging *in vitro*

To study the stability of NB_{CCM} *in vitro*, we monitored changes in non-linear signal in a flow phantom where NBs were recirculated for 3 h after formation (Figure S1). The signal magnitude rapidly increased after NB injection both in B-mode and contrast mode. Non-linear signal reached a peak intensity between 10 min and 30 min after injection and subsequently decreased progressively with longer circulation times. Nevertheless, the relatively slow washout rate (-26%/h) allowed for an optimal imaging window of about 1 h and 30 min, indicating good stability of NBs. Furthermore, US irradiation with a high mechanical index indicated the capacity of circulating NBs to enhance US signal even after 3 h. Owing to their lower density compared to water, buoyancy of both NB types resulted in a hyper signal on the top of the channel. However, the higher flow rates in most healthy organs in mice should eliminate any potential buoyancy associated bubble accumulation in the vasculature.

Supplementary Figures

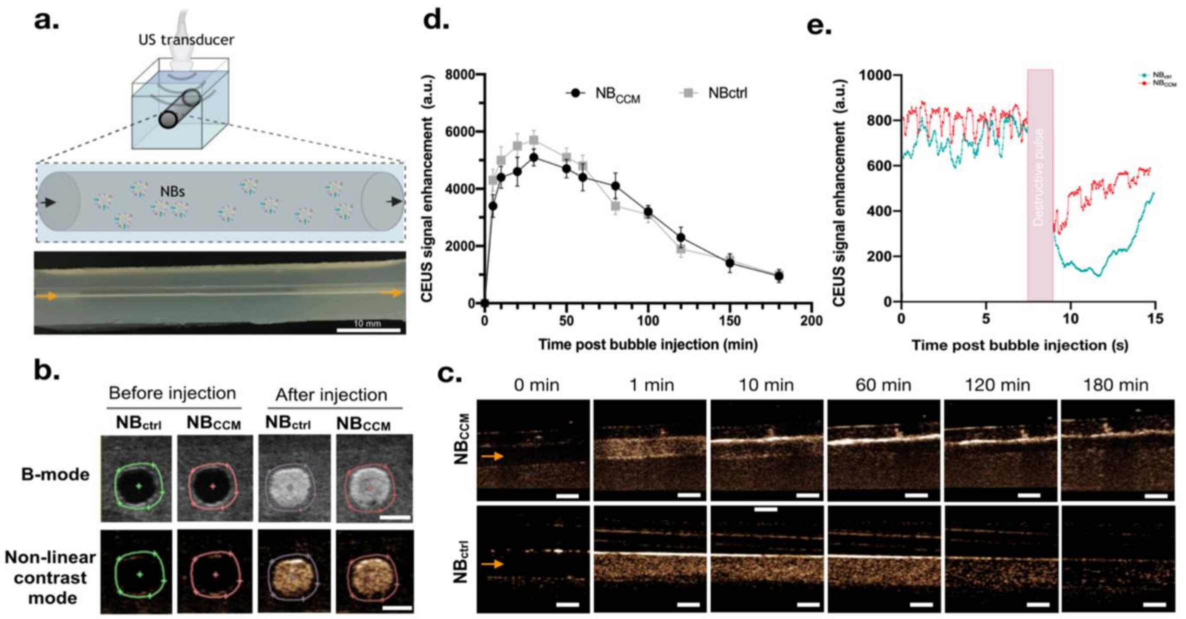

Figure S1. Stability of NBs evaluated by continuous circulation *in vitro*. (**a**) Schematic representation of the phantom setup used for assessing NB_{CCM} and NB_{ctrl} stability under US (top), and flow phantom photograph (bottom) where orange arrows represent the sample flow inside the channel; (**b**) Representative US images of two channel transverse sections before and after NB_{CCM} and NB_{ctrl} injection. Scale bar = 2 mm; (**c**) Longitudinal channel section imaging before injection and 1, 10, 60, 120 and 180 min after. Orange arrows indicate the middle of the channel before injection. Scale bar = 2 mm; (**d**) Corresponding time intensity curves. Data are shown as Mean ± SD. (**e**) Replenishment of non-linear signals after destructive pulse.

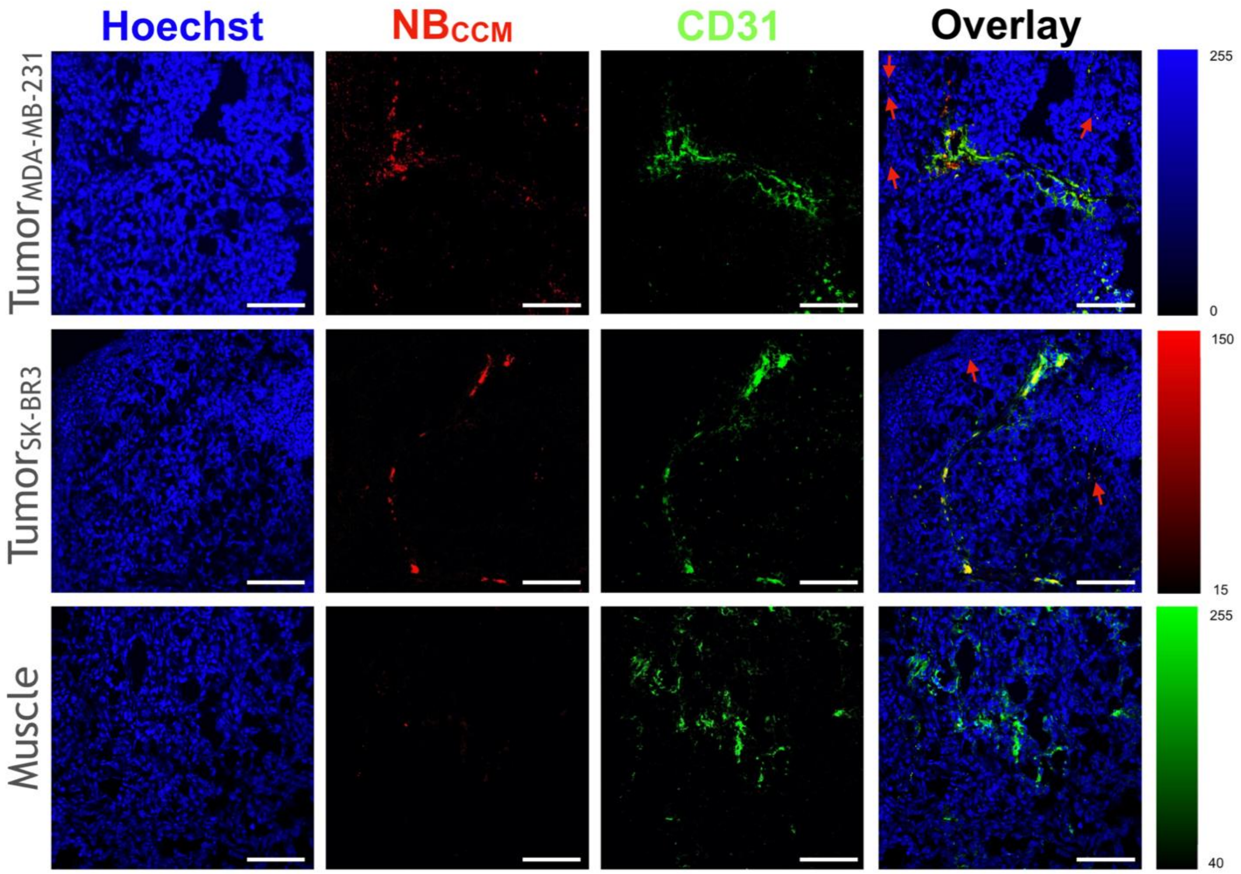

Figure S2. Representative fluorescence images of ICG-NB$_{CCM}$ 24 h after injection. Tumor and adjacent skeletal muscle sections were stained for CD31 (green) and Hoechst (blue) for visualization of blood vessels and nuclei, respectively. Red arrows indicate the position of extravascular ICG-NB$_{CCMs}$. Scale bar = 100 µm

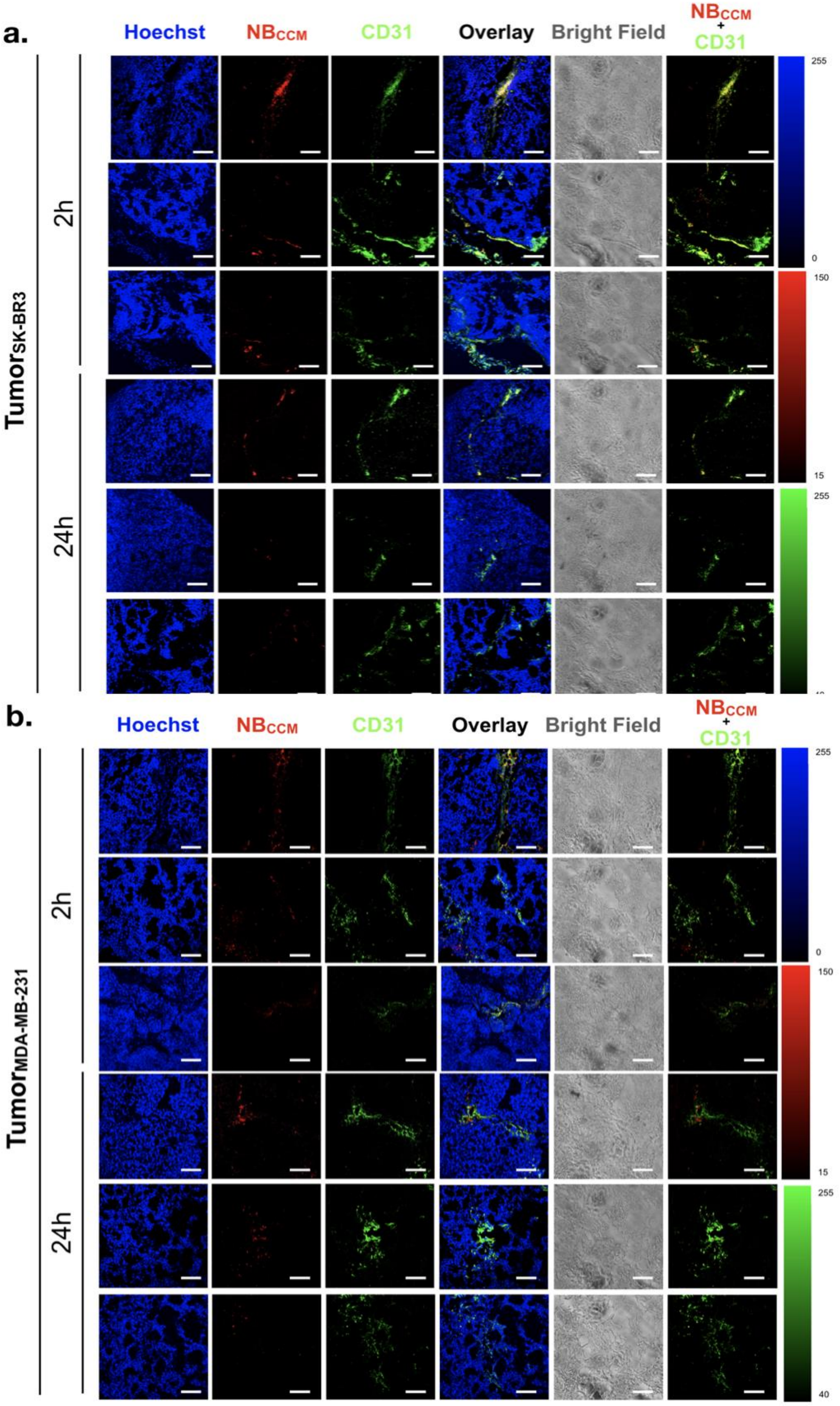

Figure S3. Confocal fluorescence microscopic images of each NB_{CCM}-treated animal, 2 or 24 h post-injection. Scale bar = 100 µm.

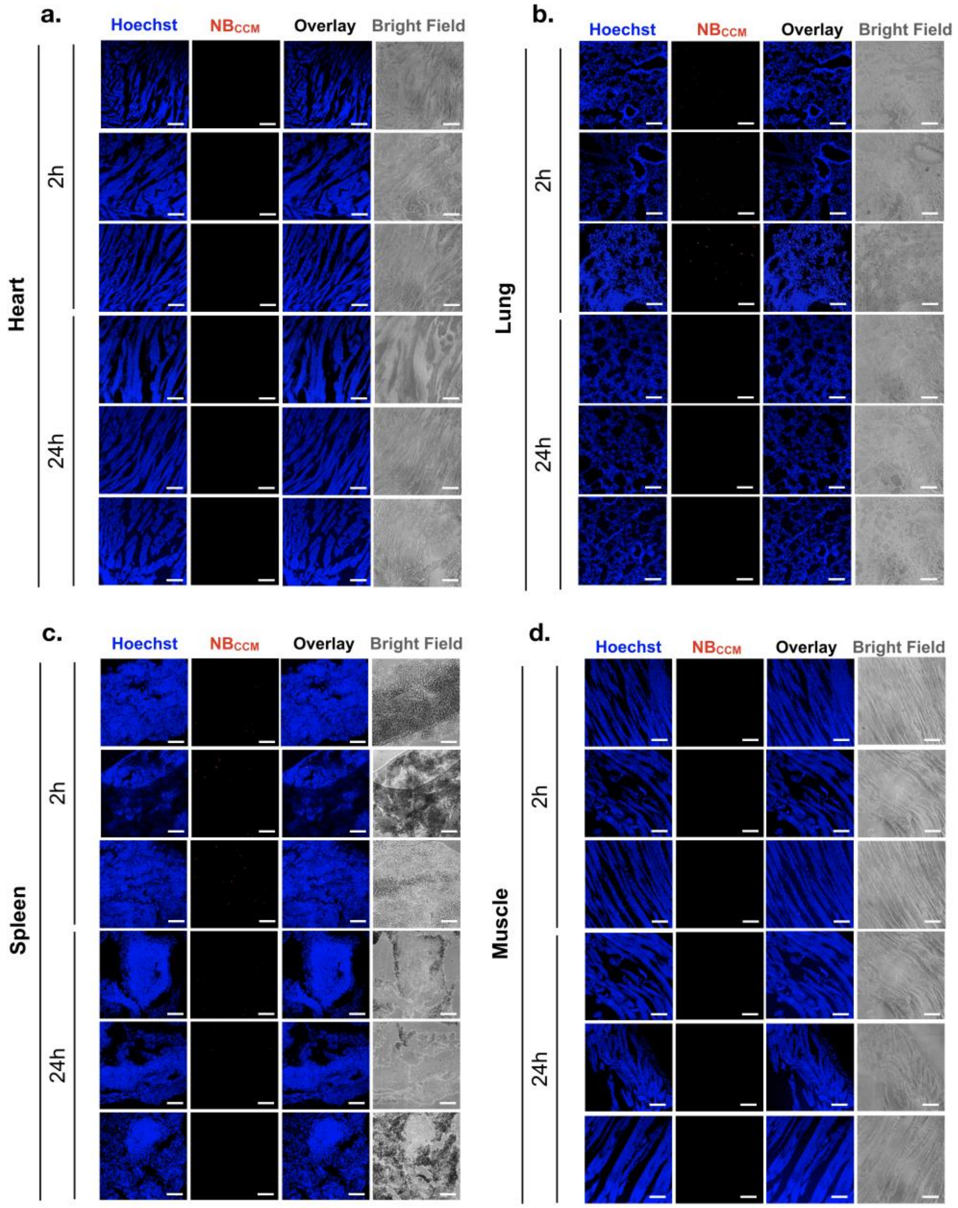
a.
Hoechst
NBCCM
Overlay
Bright Field
Heart
2h
24h
b.
Hoechst
NBCCM
Overlay
Bright Field
Lung
2h
24h
c.
Hoechst
NBCCM
Overlay
Bright Field
Spleen
2h
24h
d.
Hoechst
NBCCM
Overlay
Bright Field
Muscle
2h
24h

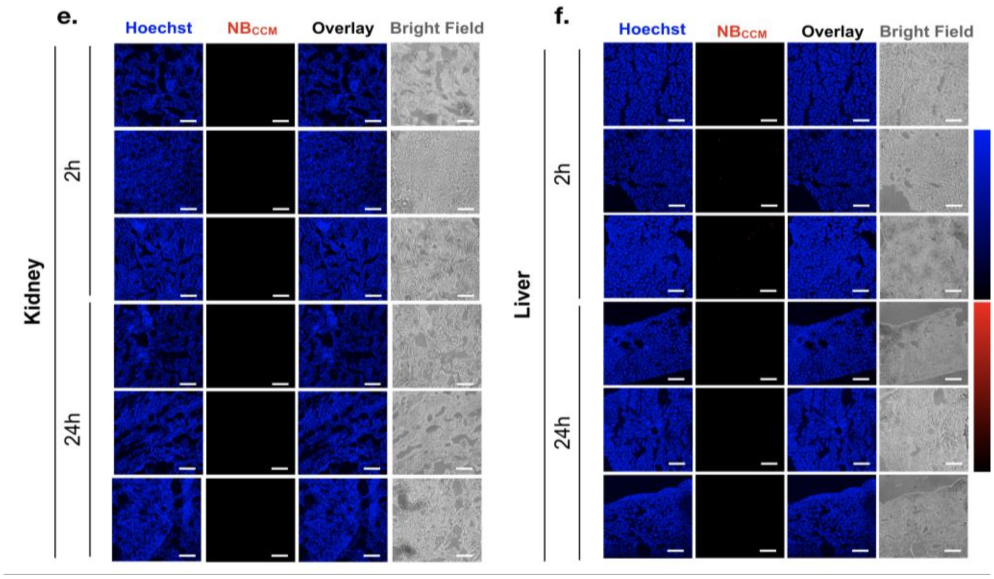

Figure S4. Confocal fluorescence microscopic images of each NB_{CCM}-treated animal, 2 or 24 h post-injection, for **(a)** heart, **(b)** lung, **(c)**, spleen, **(d)** muscle, **(e)** kidney, **(f)** liver. Scale bar = 100 μm.

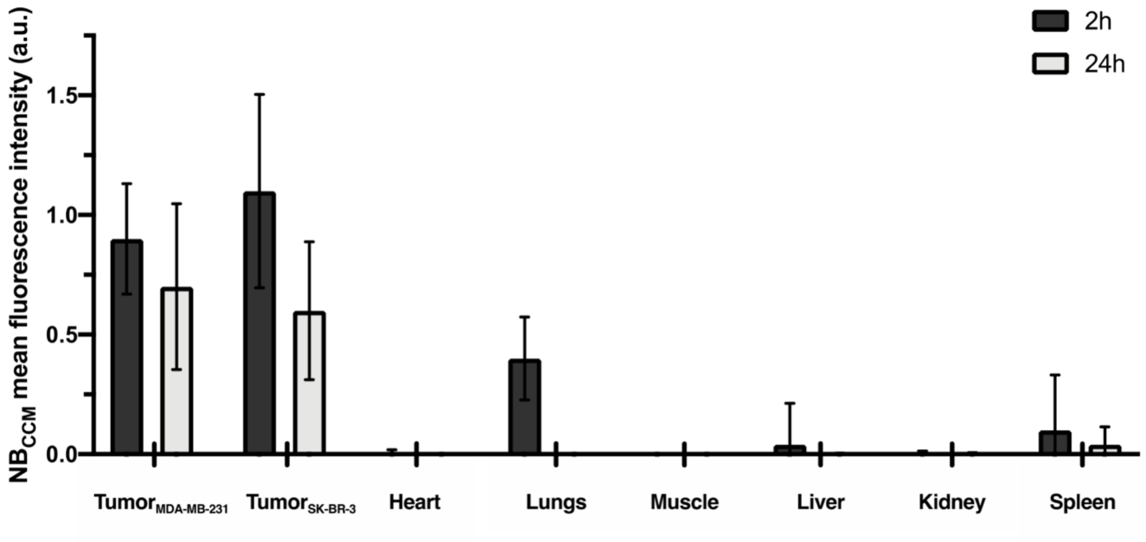

Figure S5. NB$_{CCM}$ biodistribution in different organs and tissues (lungs, heart, liver, spleen, kidneys, muscle, and tumors) using quantitative fluorescence imaging. Data are shown as mean ± SEM; (n= 3).
